# A Rare Case of Idiopathic Megacolon and Megarectum

**DOI:** 10.7759/cureus.75229

**Published:** 2024-12-06

**Authors:** Saumyaa Vohra, Aryan Gulati, Atif Alvi

**Affiliations:** 1 General Surgery, Gulf Medical University, Ajman, ARE; 2 General Surgery, King’s College Hospital London, Dubai Hills, Dubai, ARE

**Keywords:** abdominal distention, acute abdomen, constipation, idiopathic megacolon, idiopathic megarectum, stool impaction

## Abstract

Idiopathic megacolon and megarectum are rare clinical conditions characterized by irreversible dilation of the colon and rectum without an identifiable organic cause. The underlying pathophysiology remains poorly understood, though hypotheses suggest abnormalities in the enteric nervous system or smooth muscle dysfunction. These conditions present significant diagnostic and therapeutic challenges, especially in cases refractory to conservative treatment.

We present a case of a 19-year-old male with chronic constipation, progressive abdominal distension, and weight loss over one year, culminating in bowel obstruction. Imaging revealed a massively dilated sigmoid colon and rectum, measuring up to 80 cm in length and filled with approximately 10 kg of fecal matter. The patient underwent a two-stage surgical intervention, including a Hartmann’s procedure with sigmoid colectomy and a subsequent completion proctectomy with coloanal anastomosis following symptom recurrence. Histopathological evaluation confirmed the presence of ganglion cells, ruling out Hirschsprung's disease and establishing the diagnosis of idiopathic megacolon and megarectum. Postoperative complications, including a pelvic abscess and surgical site infection, were effectively managed with antibiotics and percutaneous drainage.

This case highlights the complexity of diagnosing and managing idiopathic megabowel and emphasizes the importance of individualized surgical interventions. Furthermore, it emphasizes the need for long-term follow-up and calls for further genetic and histological studies to explain the underlying mechanisms and establish standardized management protocols for this challenging condition.

## Introduction

Idiopathic megacolon and megarectum, collectively referred to as idiopathic megabowel, represent a rare clinical entity characterized by pronounced dilation of the colon and rectum in the absence of identifiable organic causes [1].

Despite the scarcity of large-scale studies establishing its precise incidence, this condition presents significant challenges in diagnosis and management due to a lack of comprehensive understanding of its pathophysiology. However, hypotheses associated with certain abnormalities in the enteric nervous system or smooth muscle of the gastrointestinal tract are being considered [2]. The pathogenesis of idiopathic megacolon and megarectum involves a complex interplay of factors leading to loss of intestinal peristalsis and subsequent dilation of the colon and rectum. This results in clinical manifestations that present similarly to other gastrointestinal disorders, including chronic constipation, abdominal distention, and recurrent abdomen pain, making the diagnosis primarily one of exclusion [3].

Managing idiopathic megacolon and megarectum is difficult since conservative treatments, like laxatives and enemas, are commonly inadequate to address the condition's severity, especially in cases of advanced dilation and obstruction of the colon. Surgical interventions, such as colectomy or proctectomy, remain the cornerstone of treatment in severe cases. However, the lack of established treatment guidelines in the current literature adds an additional layer of complexity to clinical decision-making, highlighting the need for further research efforts in this area.

Here, we present a rare and complex case of a young 19-year-old male with idiopathic megacolon and megarectum - a combination seldom reported in scientific literature. The patient’s condition was marked by extreme colonic and rectal dilation, with the sigmoid colon measuring 80 cm in length, 15-18 cm in width, and containing approximately 10 kg of fecal matter. The patient underwent successful management with a two-stage surgery, including a sigmoid colectomy with end colostomy formation, followed by a completion proctectomy, highlighting the importance of timely surgical intervention in certain cases of this challenging condition. This report highlights the diagnostic and therapeutic challenges associated with idiopathic megabowel and contributes to the growing body of literature on the importance of individualized surgical approaches. It further emphasizes the need for continued research to better understand the underlying mechanisms and develop standardized management protocols for this complex condition.

## Case presentation

A 19-year-old male presented with a one-year history of severe chronic constipation, abdominal distension, abdominal pain, nausea, and progressive weight loss, which had worsened over the preceding months. The patient described localized abdominal pain, predominantly in the epigastric and umbilical regions, that worsened after meals. Constipation was severe, with bowel movements occurring only once every three to four weeks. Over the past year, the patient reported a significant unintentional weight loss of 10 kg. His medical history included intermittent constipation since childhood, with bowel movements occurring once every four to five days. There was no significant family history of bowel disorders.

**Figure 1:**
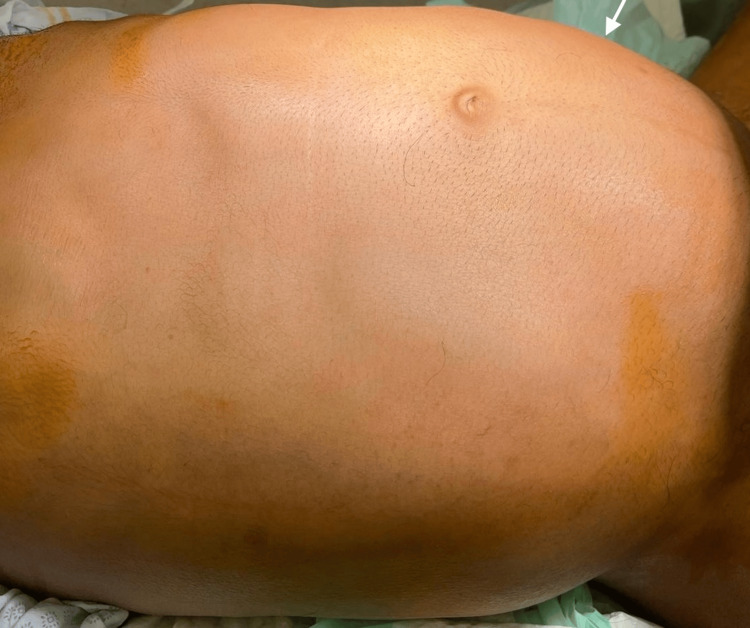
Asymmetric distension of the abdomen on physical examination. The asymmetric enlargement of the abdomen involved all quadrants of the abdomen, with notable prominence in the left hypochondriac, left lumbar, umbilical, and left iliac region.

On physical examination, the patient appeared normotensive, vitally stable, and malnourished, with a grossly distended abdomen. The distension was asymmetric, involving multiple abdominal regions, with notable prominence in the left hypochondriac, left lumbar, umbilical, and left iliac regions (Figure 1). Upon palpation, a hard abdominal mass was felt predominantly in the left upper, left lower, and right lower quadrants, which was dull on percussion. In contrast, the right upper quadrant felt soft and resonant on percussion. A digital rectal examination confirmed hard stool impaction.

**Figure 2:**
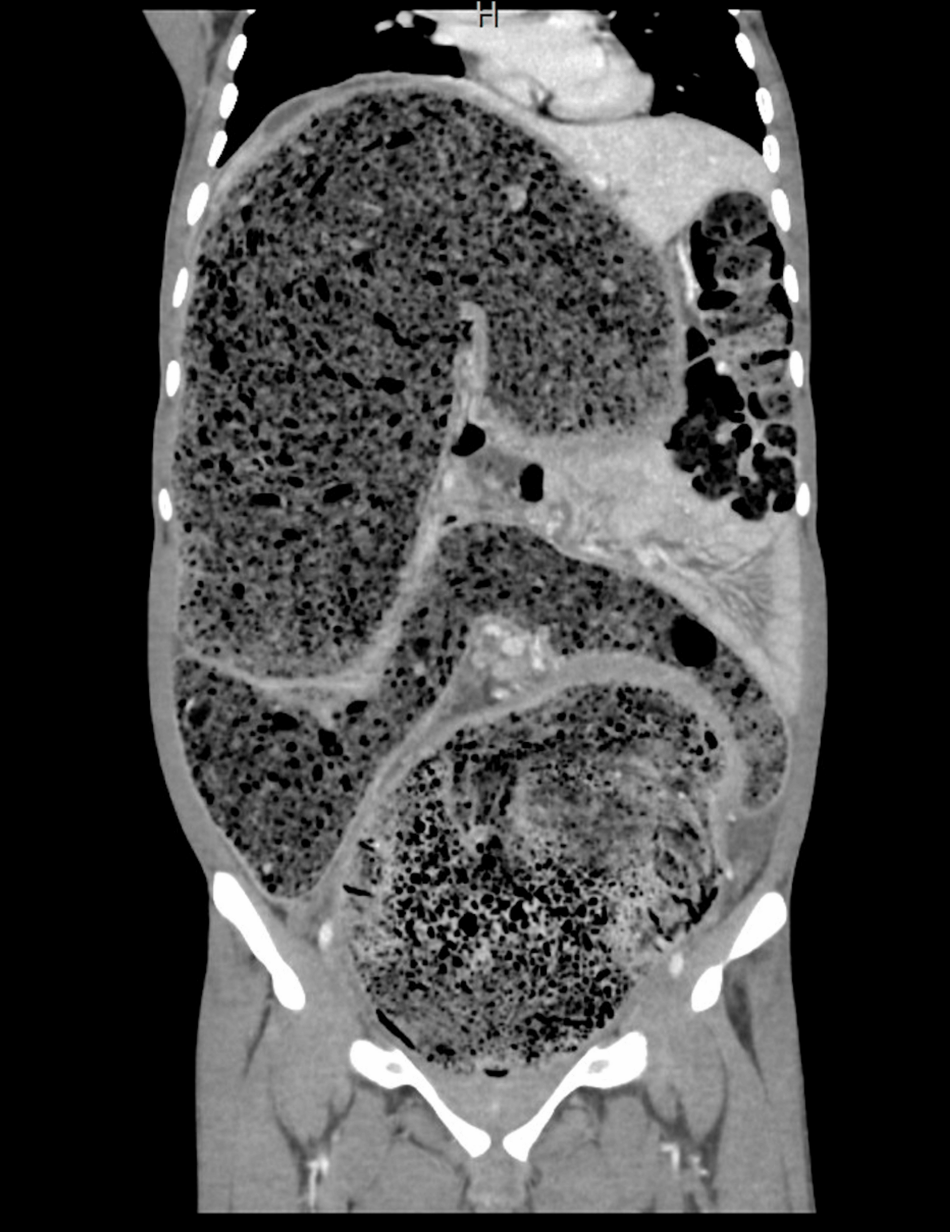
Coronal CT scan with contrast displaying colon fully loaded with feces. The colon is 15-18 cm in width and 60-80 cm in length. This is an image of a huge dilated colon that is loaded with feces.

**Figure 3:**
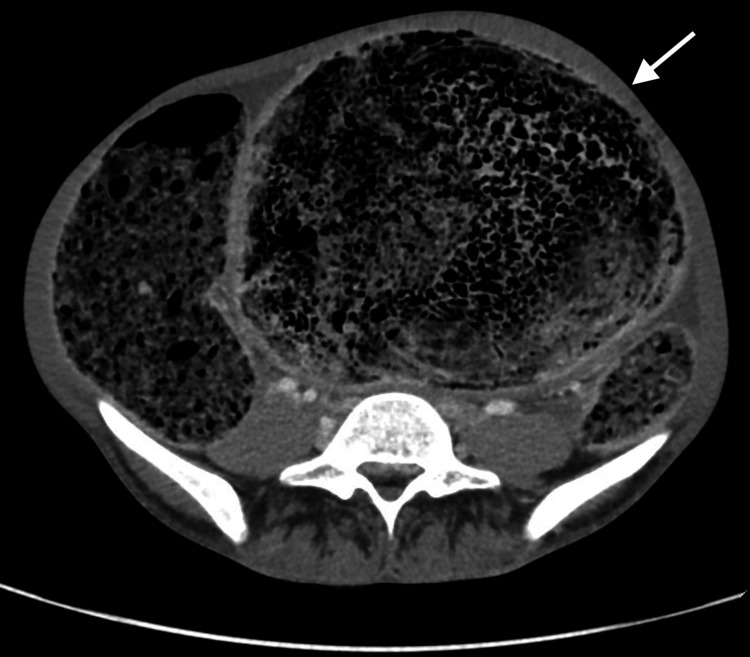
Axial CT scan with contrast displaying colon fully loaded with feces. The colon is 15 cm in width and 60-80 cm in length. This is an image of a huge dilated sigmoid colon that is loaded with feces.

All of his laboratory investigations were unremarkable. Contrast-enhanced CT imaging of the abdomen revealed a markedly dilated sigmoid colon and rectum filled with fecal matter, with a maximum colonic diameter of 15-18 cm (Figures 2, 3).

The patient was admitted and treated aggressively with intravenous fluids, oral and rectal laxatives, suppositories, and enemas, but these measures failed to relieve the impacted fecal matter. The patient continued to experience persistent abdominal pain and an inability to pass stool. As a result, an emergency laparotomy with bowel resection was deemed necessary.

**Figure 4:**
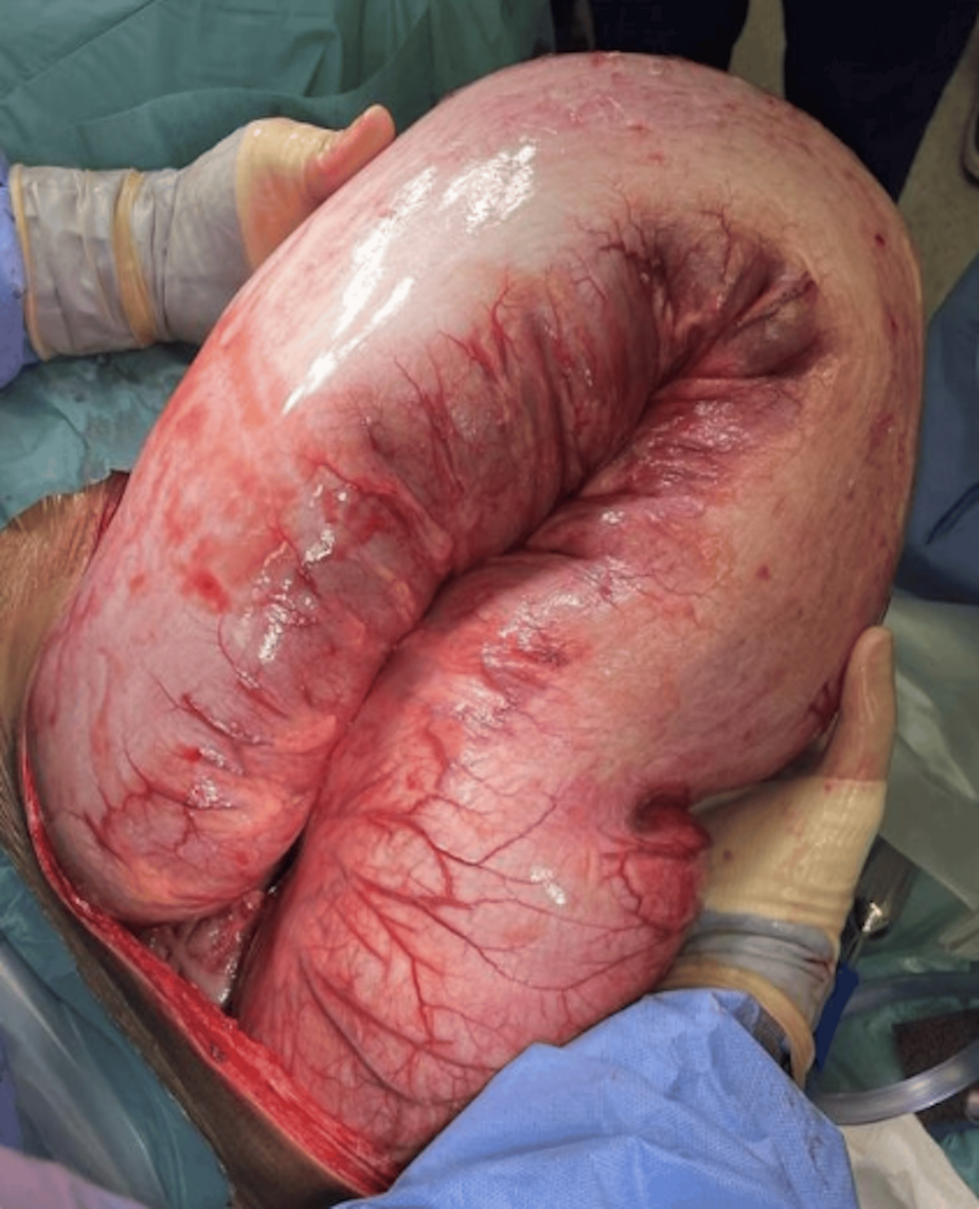
Intraoperative findings showing massive dilatation of the sigmoid colon.

**Figure 5:**
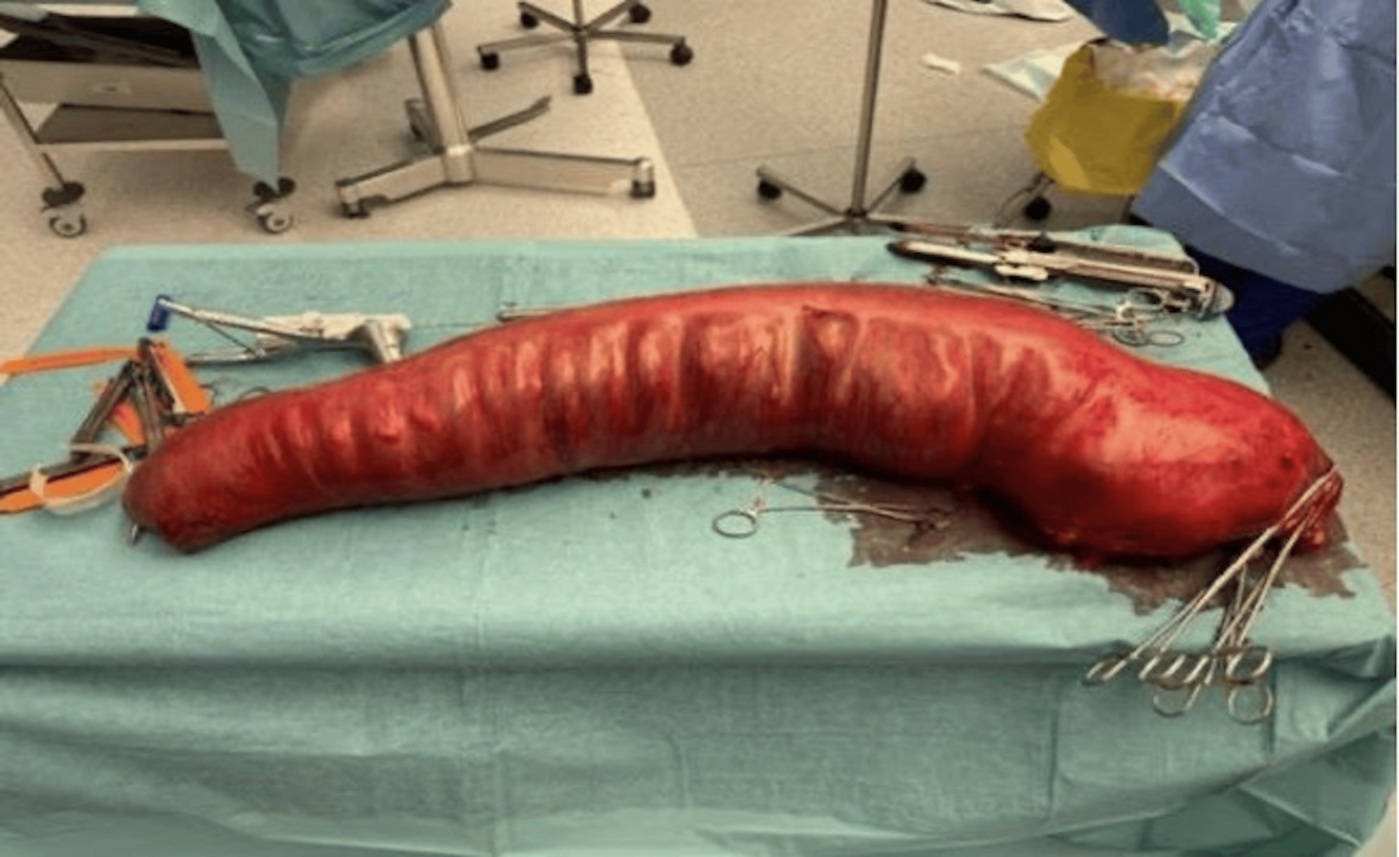
Intraoperative findings demonstrating specimen of the sigmoid colon with maximum length of 80 cm.

Intraoperative findings revealed a massively dilated sigmoid colon and rectum filled with rock-hard feces weighing approximately 10 kg and measuring 80 cm in length (Figures 4, 5). The remainder of the colon, proximal to the mid-descending segment, was distended with gas but contained no fecal matter. A Hartmann’s procedure was performed, involving resection of the dilated sigmoid colon, formation of an end colostomy, and leaving the rectal stump in situ. The surgery was carried out by a highly experienced colorectal surgeon.

Postoperatively, the patient developed a pelvic abscess, which was successfully managed with broad-spectrum antibiotics and image-guided percutaneous drainage. Additionally, a surgical site infection at the laparotomy incision site was identified and treated effectively with targeted antibiotic therapy.

Hirschsprung disease was initially considered the primary differential diagnosis. However, histopathology revealed mucosal inflammation and the presence of ganglion cells in the descending colon, sigmoid colon, and rectum, ruling out Hirschsprung disease and suggesting idiopathic megacolon as the likely diagnosis. In addition, a gross examination of the specimen revealed flattened mucosa with thinning of the walls and no polyps or ulcers, ruling out other secondary causes of megacolon. The patient subsequently underwent a colorectal anastomosis as a second-stage surgery. This resulted in good functional outcomes for one year.

**Figure 6:**
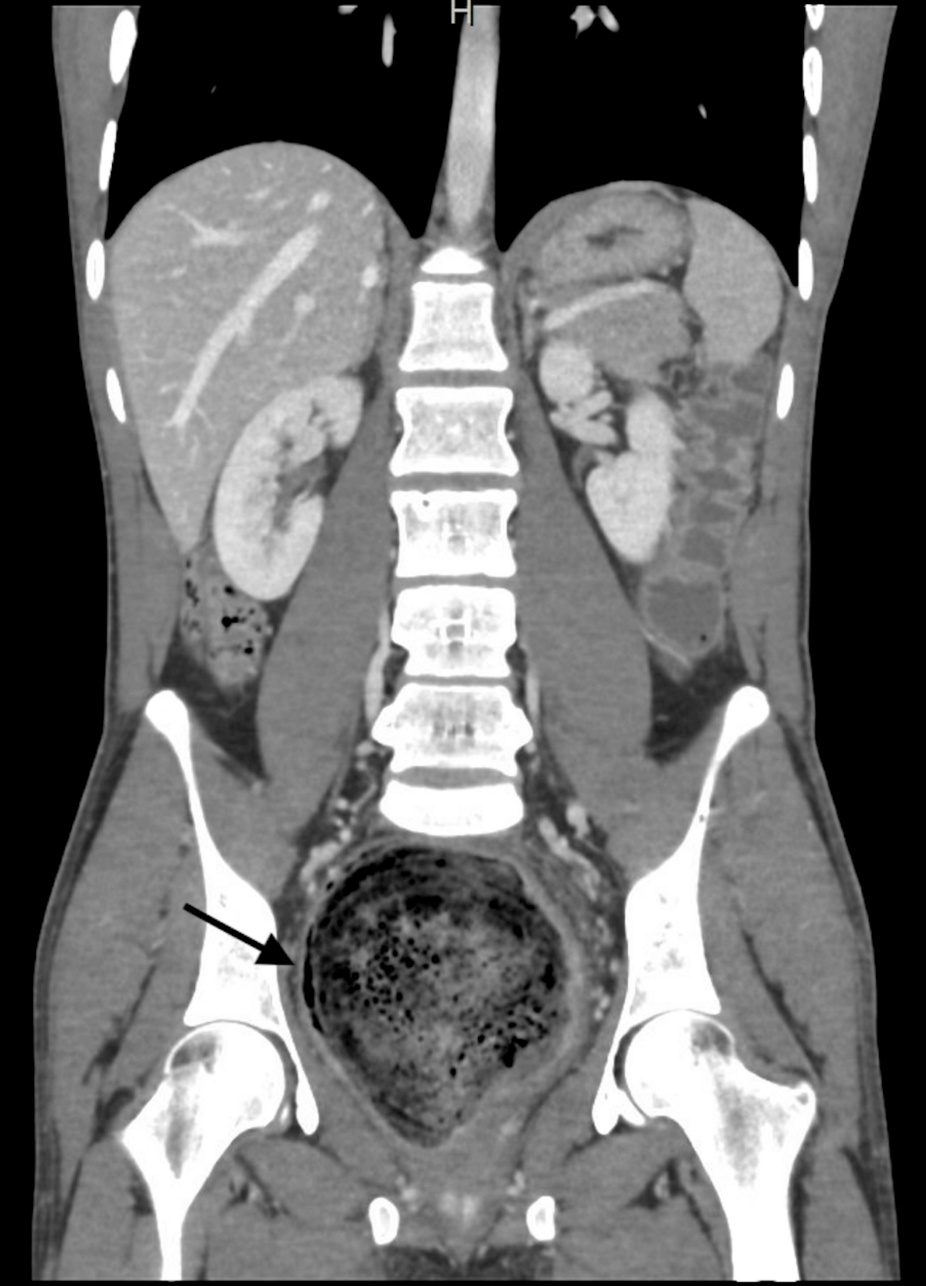
Coronal CT scan with contrast showing significant focal dilatation of the rectum with a large fecaloma inside, measuring about 12.2 cm in length, 8 cm in width, and 10 cm in height. This is an image of a huge dilated rectum that is loaded with feces.

**Figure 7:**
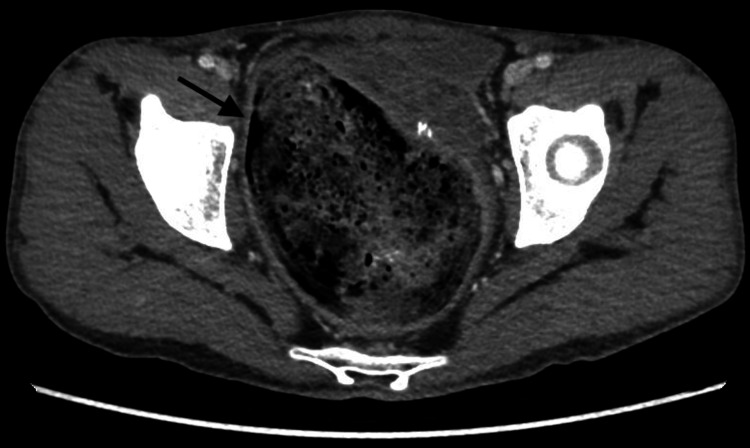
Axial CT scan with contrast showing significant focal dilatation of the rectum with a large fecaloma inside, measuring about 12.2 cm in length, 8 cm in width, and 10 cm in height. Image of a huge dilated rectum loaded with feces.

A year later, the patient presented with another bout of constipation and hard stool impaction on digital rectal examination. A CT scan with contrast revealed significant focal dilatation of the rectum with a large fecaloma inside, measuring about 12.2 cm in length, 8 cm in width, and 10 cm in height, below the colorectal anastomosis (Figures 6, 7). Megarectum was concluded as the final diagnosis.

Similar to the previous episode, the patient was not responding to conservative therapy. Therefore, the patient underwent surgery for complete proctectomy and coloanal anastomosis. Once again, histopathology revealed the presence of ganglion cells in the rectum, suggesting idiopathic megacolon as the likely diagnosis. The postoperative period after proctectomy was unremarkable.

Upon follow-up, the patient has recovered and is feeling well. The patient has now resumed his daily activities and work.

## Discussion

Idiopathic megacolon and megarectum are characterized by significant distention of the colon and rectum without any discernible etiology. This condition presents significant challenges in diagnosis and management due to its nonspecific presentation and the limited understanding of its underlying pathophysiology. Patients with idiopathic megacolon have variable presentation, including constipation or increased bowel frequency, pain, and variable need for laxatives. On the other hand, all patients suffering from idiopathic megarectum have findings suggestive of soiling and stool impaction [4]. All these symptoms are common among other gastrointestinal pathologies and thus become a difficult diagnosis since there are limited clinical features that are specific to the condition. Severe cases, such as the one presented in this report, may progress to bowel obstruction. Therefore, early diagnosis and intervention are critical to mitigate complications and implement appropriate treatment strategies, given that delayed surgical intervention may result in life-threatening consequences such as perforation-induced peritonitis, electrolyte disturbances due to fluid shifts, and sepsis [5].

The underlying pathophysiology of idiopathic megacolon and megarectum remains incompletely understood. Current hypotheses suggest potential abnormalities in the enteric nervous system or smooth muscle of the gastrointestinal tract, leading to impaired motility and subsequent colonic dilation. Secondary changes such as muscular hypertrophy and fibrosis are often observed because of chronic dilation. A recent study has investigated different genetic associations with chronic acquired megacolon and suggested a central role of the SEMA3F gene in the etiology of this condition, though further investigations are warranted to validate this hypothesis [6].

The diagnosis of idiopathic megacolon and megarectum relies on a systematic approach to exclude secondary causes, including Hirschsprung’s disease, Chagas disease, and mechanical obstruction. Imaging plays a pivotal role, with modalities like CT scans and barium enema studies providing critical information on the extent of colonic dilation and identifying complications such as perforation or fecal impaction. In this case, CT findings revealed significant colonic dilation, consistent with idiopathic megabowel. Diagnostic criteria for idiopathic megacolon have evolved, with early studies using double contrast barium enema establishing normal recto-sigmoid diameters under 6.5 cm. Idiopathic megacolon is primarily diagnosed by imaging, based on colon diameter exceeding 6.5 cm at the pelvic loop, 8 cm in the ascending colon, and 12 cm at the cecum [7]. However, these measurements vary between studies. Some propose a 10-cm threshold with variations of 2-3.5 cm, and others suggest criteria like the location of the sigmoid loop or the persistence of symptoms after surgery. CT offers a more precise assessment of colon dimensions, wall thickness, edema, and inflammation.

Although histopathology findings are not included as part of the diagnostic criteria, it is critical to exclude alternate diagnoses. In this case, gross examination of the sigmoid and upper rectum demonstrated thinning of the wall and flattening of the mucosa with no ulcers or polyps. Histopathology of the specimen confirmed mucosal inflammation and the presence of ganglion cells, ruling out other differential diagnoses like Hirschsprung’s disease and supporting the diagnosis of idiopathic megacolon. Similarly, Gattuso et al. found that the architecture of the enteric innervation is intact in resected tissues of patients with idiopathic megarectum. In addition, there was no alteration in the density of innervation in patients with idiopathic megacolon [8].

Management of idiopathic megacolon and megarectum remains challenging, particularly in patients refractory to conservative measures such as laxatives, enemas, and dietary modifications. Surgical intervention is often necessary, particularly in cases involving severe dilation, recurrent obstruction, or intractable symptoms. In this case, the patient underwent sigmoid colectomy and completion proctectomy, leading to significant symptomatic relief and functional recovery. Surgical options vary depending on the extent of colonic involvement and include segmental colectomy, total proctocolectomy with ileostomy, or restorative proctocolectomy. Gladman et al. support this intervention, recommending colectomy as the optimum procedure in patients with non-dilated rectum with a success rate of 71.1%. Meanwhile, restorative proctocolectomy is suggested to treat dilation of the colon and rectum, despite proctectomy, the Duhamel, and pull-through procedures associated with significant mortality (3-25%) [9].

Postoperative outcomes can vary, and complications such as pelvic abscess, surgical site infection, anastomotic leaks, and nutritional deficiencies are well-documented risks, especially following extensive bowel resections. In this case, a pelvic abscess and surgical site infection were managed successfully with broad-spectrum antibiotics and image-guided drainage, emphasizing the importance of close postoperative monitoring. Studies have demonstrated that delayed surgical intervention in idiopathic megacolon can lead to serious complications such as perforation, sepsis, and electrolyte disturbances, further supporting the need for timely surgical intervention in refractory cases [5].

This case highlights the complexity of diagnosing and managing idiopathic megacolon and megarectum. It emphasizes the critical role of thorough diagnostic evaluation, including imaging and histopathology, in ruling out secondary causes. The successful surgical management of this patient highlights the importance of individualized treatment strategies and timely intervention in achieving favorable outcomes. Additionally, the absence of clear treatment guidelines in the literature reflects the need for further research into the underlying mechanisms and long-term outcomes of idiopathic megacolon and megarectum. Larger cohort studies and ongoing investigations into potential genetic and histopathological contributors are essential to establish standardized diagnostic criteria and treatment protocols for this rare but clinically significant condition.

## Conclusions

Idiopathic megacolon and megarectum are an unusual cause of acute bowel obstruction and are primarily diseases of exclusion. The presence of ganglionic cells within the plexus of the colon and rectum excludes the diagnosis of Hirschsprung disease and suggests the diagnosis of idiopathic megacolon and megarectum. This condition is difficult to treat with conservative management alone. Therefore, in most cases, such as this one, surgical intervention is the only way to resolve the condition. However, due to a lack of scientific literature and the absence of standardized treatment guidelines with regard to this disease, more research should be warranted, especially given the complexity of managing such a condition.
